# The Diversity of the CRISPR-Cas System and Prophages Present in the Genome Reveals the Co-evolution of *Bifidobacterium pseudocatenulatum* and Phages

**DOI:** 10.3389/fmicb.2020.01088

**Published:** 2020-05-26

**Authors:** Gang Wang, Qian Liu, Zhangming Pei, Linlin Wang, Peijun Tian, Zhenmin Liu, Jianxin Zhao, Hao Zhang, Wei Chen

**Affiliations:** ^1^State Key Laboratory of Food Science and Technology, Jiangnan University, Wuxi, China; ^2^State Key Laboratory of Dairy Biotechnology, Shanghai Engineering Research Center of Dairy Biotechnology, Dairy Research Institute, Bright Dairy & Food Co., Ltd., Shanghai, China; ^3^School of Food Science and Technology, Jiangnan University, Wuxi, China; ^4^International Joint Research Laboratory for Probiotics, Jiangnan University, Wuxi, China; ^5^(Yangzhou) Institute of Food Biotechnology, Jiangnan University, Yangzhou, China; ^6^National Engineering Research Center for Functional Food, Jiangnan University, Wuxi, China; ^7^Wuxi Translational Medicine Research Center and Jiangsu Translational Medicine Research Institute Wuxi Branch, Wuxi, China; ^8^Beijing Innovation Centre of Food Nutrition and Human Health, Beijing Technology and Business University, Beijing, China

**Keywords:** *Bifidobacterium pseudocatenulatum*, CRISPR-Cas systems, prophage, co-evolution, genomic diversity

## Abstract

Diverse CRISPR-Cas systems constitute an indispensable part of the bacterial adaptive immune system against viral infections. However, to escape from this immune system, bacteriophages have also evolved corresponding anti-defense measures. We investigated the diversity of CRISPR-Cas systems and the presence of prophages in the genomes of 66 *Bifidobacterium pseudocatenulatum* strains. Our findings revealed a high occurrence of complete CRISPR-Cas systems (62%, 41/66) in the *B. pseudocatenulatum* genomes. Subtypes I-C, I-U and II-A, were found to be widespread in this species. No significant association was found between the number of bacterial CRISPR spacers and its host’s age. This study on prophages within *B. pseudocatenulatum* genomes revealed that prophage genes related to distinct functional modules became degraded at different levels, indicating that these prophages were not likely to enter lytic cycle spontaneously. Further, the evolutionary analysis of prophages in this study revealed that they might be derived from different phage ancestors. Notably, self-targeting phenomenon within *B. pseudocatenulatum* and Anti-CRISPR (Acr) coding genes in prophages was observed. Overall, our results indicate that the competition between *B. pseudocatenulatum* and phages is a major driving factor for the genomic diversity of both partners.

## Introduction

Bifidobacteria are one of the earliest colonizers of the human gut and the predominant microbial group in infants and healthy adults (Arboleya et al., 2016). The abundance of bifidobacteria in the gut is often considered as an indicator of the human health status and has been proven to be correlated with various intestinal and immunological disorders, such as inflammatory bowel disease, irritable bowel syndrome, obesity and diabetes (Turroni et al., 2014). As an important member of the *Bifidobacterium* genus, *Bifidobacterium pseudocatenulatum* is commonly found in the fecal samples of human across all ages (Turroni et al., 2012) and is especially abundant in breastfeeding infants (Gore et al., 2008). Compared with other *Bifidobacterium* species, *B. pseudocatenulatum* has been shown to be significantly associated with metabolic diseases in both animal experiments (Agusti et al., 2018) and clinical trials (Wu et al., 2017; Zhao et al., 2018). In addition, some *B. pseudocatenulatum* strains have been noted for their beneficial properties, such as the production of enterolignan, urolithin and conjugated linoleic acid (Yang et al., 2017; Gaya et al., 2018; Peiroten et al., 2019). Therefore, *B. pseudocatenulatum* is considered as the next-generation probiotic species for its potential beneficial effects.

One of the main challenges for probiotics is to overcome the harsh conditions in the gastrointestinal tract. The human gut is a natural reservoir of bacteriophages and it is expected that > 10^12^ phage particles reside in the human gut (Shkoporov and Hill, 2019). Even though temperate phages are widespread (Kim and Bae, 2018), the presence of phage particles still provide a challenge for the survival of probiotics in the intestine. A major strategy for bacteria to resist bacteriophage infection is via an immune mechanism known as Clustered Regularly Interspaced Short Palindromic Repeats (CRISPR), together with CRISPR-associated Cas enzymes (Rodolphe et al., 2007). As a heritable adaptive immune system in bacteria and archaea, the CRISPR-Cas system selects foreign nucleic acids and integrates it into the CRISPR array in the form of spacer sequences to provide a memory of infection. Upon reinfection, CRISPR-Cas comes into action, deploying RNA-guided nucleases for silencing specific sequences of the foreign genetic materials. Cas proteins encoded by *cas* genes adjacent to the CRISPR array are necessary for the three phases of CRISPR-Cas immunity: adaptation, CRISPR RNA (crRNA) biogenesis and interference. During adaptation, foreign nucleic acids are captured, processed and then integrated into the CRISPR array. For retrieving the memory, this CRIPSR-spacer array is transcribed to generate a precursor crRNA (pre-crRNA) that is further processed to generate mature crRNAs. Upon subsequent infection, the interference machinery, is guided by mature crRNAs to identify the foreign invader and cleave its nucleic acid sequences, thus protecting the bacteria from infection (Hille et al., 2018).

Due to the antagonistic coevolution between bacteria and bacteriophages over billions of years, bacteriophages evolved an alternative form of infection, namely lysogeny. Under such circumstances, prophage refers to the temperate phage genome that is integrated into the host bacterial chromosome, replicating with its host without producing virion progeny (Howard-Varona et al., 2017). Comparative genomic analyses in early studies have shown that more than 50% of bacteria possess prophages (Canchaya et al., 2003) whilst recent study showed that the prevalence of prophages within murine gut microbiota is much higher (Kim and Bae, 2018). However, prophages can be activated under certain conditions, such as UV light (Guo et al., 2016) or chemicals (Goerke et al., 2006; Mavrich et al., 2018). A recent study also showed that fructose and short-chain fatty acids could promote prophage induction in *Lactobacillus reuteri* (Oh et al., 2019a), suggesting the effect of sugar metabolism on phage production in human gastrointestinal tract. Meanwhile, bacterial hosts can acquire some novel functions and become more competitive in the community. For example, a prophage in *Escherichia coli* increases its host’s resistance to antibiotics and oxidative stress (Wang et al., 2010); prophages within *Enterococcus faecalis* V583 encode platelet-binding-like proteins which were found to function in adhesion to human platelets (Matos et al., 2013); prophages in *L. reuteri* can be induced and released to kill competitor strains so as to be advantageous in intestinal niche (Oh et al., 2019b).

In this study, to investigate the competition between *B. pseudocatenulatum* and its bacteriophages, 66 *B. pseudocatenulatum* strains newly isolated from human and animal feces were subjected to *de novo* sequencing. The obtained genomic datasets were used to explore the diversity of CRISPR-Cas systems and the presence of prophages within the bacterial genomes. Our findings demonstrated the coevolution of both the host and the temperate phage, which may provide insights for classification of this species as a probiotics.

## Materials and Methods

### Isolation of *B. pseudocatenulatum* Strains

Fecal samples collected from human and animal in China were immersed in 60% glycerol and stored at −80°C. One volume of this feces–glycerol complex was then mixed with nine volumes of phosphate-buffered saline (pH = 6.5) and diluted serially. The fecal dilutions were plated on De Man, Rogosa and Sharpe (MRS) agar supplemented with 0.05% (wt/col) L-cysteine hydrochloride and 50 μg/ml mupirocin and incubated in an anaerobic chamber at 37°C for 48 h. Colonies with different morphology were selected for second plating and then sub-cultivated in MRS broth. The bacterial 16S rDNA fragment was amplified using 16S rDNA common primers to identify the species. Finally, 66 strains of *B. pseudocatenulatum* were detected and selected for further analyses (Supplementary Table S1).

### Genome Sequencing and Assemblies

The draft genomes of the 66 strains were sequenced at Majorbio Bio-pharm Technology Co. Ltd. (Shanghai, China) using Illumina Hiseq × 10 platform to achieve a sequencing coverage of at least 100-fold. Raw data were assembled with the SOAPdenovo2 software using K-mer size trials to obtain the best result. Partial assembly and optimization were performed according to the paired-end and overlap relationships to form scaffolds. The length of the average scaffold N50 is 321,717 bp, accounting for 14% total genome length. The genome sequences were submitted to NCBI database and the accession numbers can be seen in Supplementary Table S1.

### CRISPR-Cas Detection and Identification

The genomes of the 66 *B. pseudocatenulatum* strains were then subjected to CRISPR detection using the Mince software (Bland et al., 2007) and *cas* gene detection using the CRISPR-Cas++ website^1^, which was also used for subtype prediction (Couvin et al., 2018). Subsequently, manual curation was performed to check whether the strains had the important *cas* genes that play a key role in the bacterial immune function, e.g., the spacer integrase genes involved in the adaptation period; the Cas1, Cas2 (Nunez et al., 2014), and Cas3 protein genes involved in the cleavage of targeted sequence; and the Cas9 protein gene for the type II CRISPR system (Hille et al., 2018).

### Prophage Identification

The prophages within the *B. pseudocatenulatum* genomes were identified as follows: (1) The prophages were first screened by the Prophage Hunter server (Song et al., 2019). (2) The prophages in the active status with scores greater than 0.8 were selected for the next step. (3) The selected prophages were extracted, and their open reading frames (ORFs) were predicted using the Glimmer and GeneMarkS software and annotated using BLASTP v2.2.28 analysis (*e*-value cut-off of 1e^–5^) against several reference databases [NR, Swiss-Prot, String and Cluster of Orthologous Group (COG) databases]. (4) Sequence alignment was performed between the prophages and spacer sequences in the CRISPR array. The inclusion criteria for the prophages for further analyses were as follows: prophages should have *in silico* predicted scores (using the Prophage Hunter server) greater than 0.8; genome sequence length longer than 10 kb; at least 20 ORFs (Lugli et al., 2016) and one phage-associated important gene; and complete alignment with at least one spacer sequence.

## Results

### High Occurrence of CRISPR-Cas Systems in *B. pseudocatenulatum*

**FIGURE 1:**
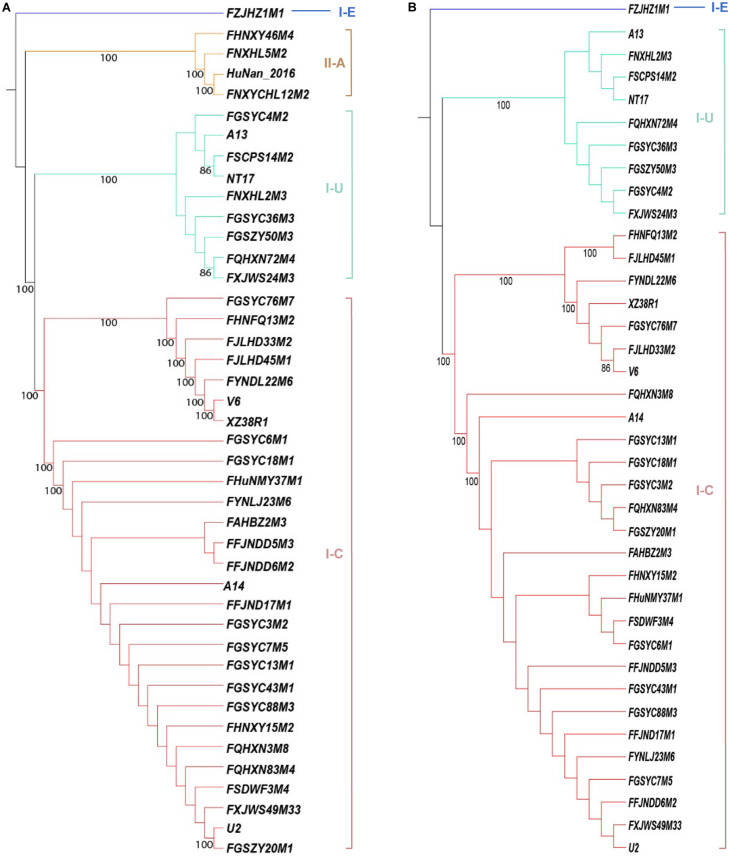
Phylogenetic tree based on the amino acid sequences of Cas proteins in *B. pseudocatenulatum*, aligned using the MUSCLE algorithm and depicted using UPGMA using 500 bootstrap replicates. Bootstrap values are represented on the nodes. The CRISPR-Cas subtypes are written on the right, and the groups are highlighted in different colors for each subtype. **(A)** Phylogenetic tree based on Cas1 amino acid sequences. **(B)** Phylogenetic tree based on Cas3 amino acid sequences.

The occurrence of CRISPR-Cas systems in *B. pseudocatenulatum* was found to be approximately 62% (41/66, Table 1). All of the 41 strains with complete CRISPR-Cas systems encoded only one *cas1* gene. Overall, 28 type I-C systems, 8 type I-U systems, 1 type I-E system and 4 type II-A systems were identified. Cas1 protein is the most conserved Cas protein and can be found in almost all CRISPR-Cas systems. The reliability of subtype classification was further confirmed by phylogenetic tree construction based on the amino acid sequences of Cas1 protein (Figure 1A). The nuclease Cas3 is the hallmark protein of type I systems (Hille et al., 2018). The subtypes within type I systems could be well distinguished in the phylogenetic tree constructed based on Cas3 amino acid sequences (Figure 1B).

**TABLE 1:** CRISPR-Cas systems present in *B. pseudocatenulatum* strains.

Strain	Type-subtype	Reapeat sequence	Repeat length	No. repeats	cas1	cas2	cas3	cas9
A13	None							
A14	I-C	GTCGCTCTCCTCATGGAGAGCGTGGATTGAAAT	33	63	Y	Y	Y	
FAHBZ2M3	I-C	GTCGCTCTCCTCATGGAGAGCGTGGATTGAAAT	33	113	Y	Y	Y	
FAHBZ9L5	None							
FAHWH24M2	None							
FFJND17M1	I-C	GTCGCTCTCCTCATGGAGAGCGTGGATTGAAAT	33	6	Y	Y	Y	
FFJND7M3	None							
FFJNDD5M3	I-C	GTCGCTCTCCTCATGGAGAGCGTGGATTGAAAT	33	83	Y	Y	Y	
FFJNDD6M2	I-C	GTCGCTCTCCTCATGGAGAGCGTGGATTGAAAT	33	91	Y	Y	Y	
FGSYC11M1	None							
FGSYC12M4	None							
FGSYC13M1	I-C	GTCGCTCTCCTCATGGAGAGCGTGGATTGAAAT	33	114	Y	Y	Y	
FGSYC18M1	I-C	GTCGCTCTCCTCATGGAGAGCGTGGATTGAAAT	33	104	Y	Y	Y	
FGSYC36M3	I-U	ATTCCTGAGCTAATCAGCTCAGGACTTCATTGAGGA	36	38	Y	Y	Y	
FGSYC39M1	None							
FGSYC3M2	I-C	GTCGCTCTCCTCATGGAGAGCGTGGATTGAAAT	33	99	Y	Y	Y	
FGSYC43M1	I-C	GTCGCTCTCCTCATGGAGAGCGTGGATTGAAAT	33	83	Y	Y	Y	
FGSYC4M2	I-U	ATTCCTGAGCTAATCAGCTCAGGACTTCATTGAGGA	36	49	Y	Y	Y	
FGSYC5M4	None							
FGSYC6M1	I-C	GTCGCTCTCCTCATGGAGAGCGTGGATTGAAAT	33	59	Y	Y	Y	
FGSYC76M7	I-C	GTCGCTCCCCGCAAGGGGAGTGTGGATTGAAAT	33	34	Y	Y	Y	
FGSYC7M5	I-C	GTCGCTCTCCTCATGGAGAGCGTGGATTGAAAT	33	80	Y	Y	Y	
FGSYC87M1	None							
FGSYC88M3	I-C	GTCGCTCTCCTCATGGAGAGCGTGGATTGAAAT	33	53	Y	Y	Y	
FGSYC91M2	None							
FGSZY20M1	I-C	GTCGCTCTCCTCATGGAGAGCGTGGATTGAAAT	33	64	Y	Y	Y	
FGSZY50M3	I-U	ATTCCTGAGCTAATCAGCTCAGGACTTCATTGAGGA	36	32	Y	Y	Y	
FHNFQ13M2	I-C	GTCGCTCCCCGCAAGGGGAGTGTGGATTGAAAT	33	23	Y	Y	Y	
FHNFQ3M1	None							
FHNXY15M2	I-C	GTCGCTCTCCTCATGGAGAGCGTGGATTGAAAT	33	90	Y	Y	Y	
FHNXY46M4	II-A	GTTTCAGATGCCTGTCAGATCAAAGACTTAGACCAC	36	13	Y			Y
FHuNMY10M3	None							
FHuNMY37M1	I-C	GTCGCTCTCCTCATGGAGAGCGTGGATTGAAAT	33	97	Y	Y	Y	
FJLHD2M3	None							
FJLHD33M2	I-C	GTCGCTCCCCGCAAGGGGAGTGTGGATTGAAAT	33	31	Y	Y	Y	
FJLHD45M1	I-C	GTCGCTCCCCGCAAGGGGAGTGTGGATTGAAAT	33	21	Y	Y	Y	
FJLHD4M2	None							
FJSNT36M3	None							
FJSNT37M5	None							
FNMHLBE12M7	None							
FNXHL2M3	I-U	ATTCCTGAGCTAATCAGCTCAGGACTTCATTGAGGA	36	25	Y	Y	Y	
FNXHL5M2	II-A	GTTTCAGATGCCTGTCAGATCAAAGACTTAGACCAC	36	13	Y			Y
FNXYCHL12M2	II-A	GTTTCAGATGCCTGTCAGATCAAAGACTTAGACCAC	36	31	Y			Y
FQHXN112M3	None							
FQHXN3M8	I-C	GTCGCTCTCCTCATGGAGAGCGTGGATTGAAAT	33	64	Y	Y	Y	
FQHXN5M4	None							
FQHXN6M4	None							
FQHXN72M4	I-U	ATTCCTGAGCTAATCAGCTCAGGACTTCATTGAGGA	36	8	Y	Y	Y	
FQHXN83M4	I-C	GTCGCTCTCCTCATGGAGAGCGTGGATTGAAAT	33	116	Y	Y	Y	
FQHXN8M3	None							
FSCPS14M2	I-U	ATTCCTGGGCTAATCAGCTCAGGACTTCATTGAGGA	36	32	Y	Y	Y	
FSDWF3M4	I-C	GTCGCTCTCCTCATGGAGAGCGTGGATTGAAAT	33	111	Y	Y	Y	
FSHXXA2M9	None							
FXJKS15M4	None							
FXJWS24M3	I-U	ATTCCTGAGCTAATCAGCTCAGGACTTCATTGAGGA	36	48	Y	Y	Y	
FXJWS49M33	I-C	GTCGCTCTCCTCATGGAGAGCGTGGATTGAAAT	33	81	Y	Y	Y	
FYNDL22M6	I-C	GTCACTCCCCGCAAGGGGAGTGTGGATTGAAAT	33	16	Y	Y	Y	
FYNLJ23M6	I-C	GTCGCTCTCCTCATGGAGAGCGTGGATTGAAAT	33	76	Y	Y	Y	
FZJHZ1M1	I-E	GTGTTCCCCGCATACGCGGGGATGATCCC	29	168	Y	Y	Y	
FZJHZD11M4	None							
HuNa38	None							
HuNan_2016	II-A	GTTTCAGATGCCTGTCAGATCAAAGACTTAGACCAC	36	47	Y			Y
NT17	I-U	ATTCCTGAGCTAATCAGCTCAGGACTTCATTGAGGA	36	41	Y	Y	Y	
U2	I-C	GTCGCTCTCCTCATGGAGAGCGTGGATTGAAAT	33	60	Y	Y	Y	
V6	I-C	GTCGCTCCCCGCAAGGGGAGTGTGGATTGAAAT	33	22	Y	Y	Y	
XZ28R1	I-C	GTCGCTCCCCGCAAGGGGAGTGTGGATTGAAAT	33	16	Y	Y	Y	

**FIGURE 2:**
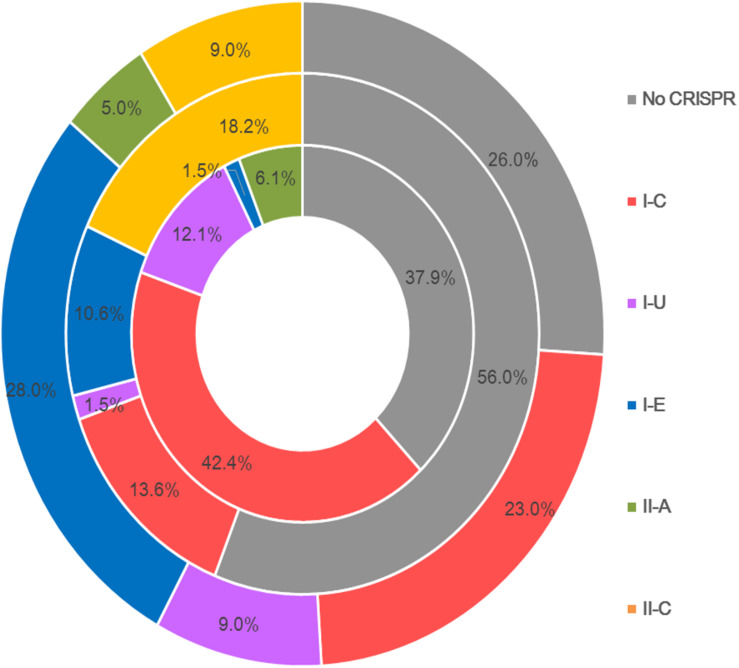
Comparison of the occurrence of CRISPR-Cas subtypes between the Bifidobacterium genus (outer circle), *B. longum* (intermediate circle) and *B pseudocatenulatum* (the innermost circle).

Type I CRISPR-Cas systems were relatively common in *B. pseudocatenulatum*. Among the subtypes of type I systems, subtype I-C showed the highest prevalence, with a coverage of 42.4%, which is much higher than its prevalence in *Bifidobacterium longum* and across the *Bifidobacterium* genus (13.6 and 23.0%, respectively) (Figure 2). Subtype I-U was found to be the second most prevalent CRIPSR-Cas system in *B. pseudocatenulatum*, although its prevalence has been reported to be only at 1.5% in *B. longum* (Hidalgo-Cantabrana et al., 2017). Type II systems were less common in *B. pseudocatenulatum*. Subtype II-C was absent in this species, whereas it is frequently found in *B. longum*. In contrast, subtype II-A was the only subtype present in *B. pseudocatenulatum*, which is absent in *B. longum* strains (Figure 2).

### CRISPR Loci Characterization in *B. pseudocatenulatum*

**FIGURE 3:**
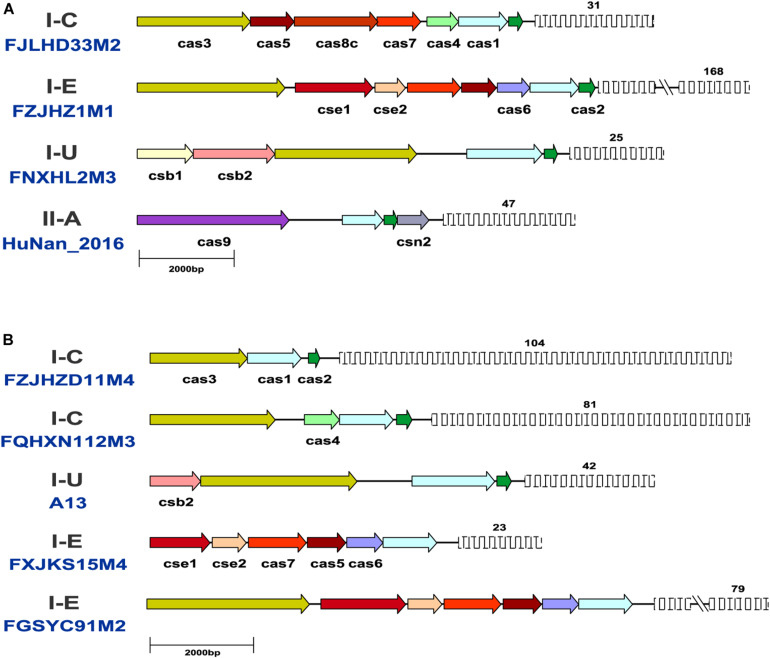
Schematic representation of CRISPR-Cas systems in *B. pseudocatenulatum*. **(A)** Representative CRISPR-Cas locus architecture of *B. pseudocatenulatum*. The same color arrow represents the same *cas* genes, and the length of the arrow represents the length of the *cas* gene; the fence graphic represents the CRISPR loci, and the upper number represents the number of repeats. Long repeat-spacer arrays were shortened for simplicity indicated by a double line break; **(B)** Schematic diagram of incomplete CRISPR-Cas systems.

The same CRISPR-Cas subtypes showed similar *cas* gene arrangement, size and direction across the *B. pseudocatenulatum* strains, but the length of the CRISPR arrays varied. The representative strains for each subtype were selected to map their CRISPR-Cas systems (Figure 3A). Compared with other reported *Bifidobacterium* CRISPR-Cas locus architectures (Briner et al., 2015; Hidalgo-Cantabrana et al., 2017), the *cas* gene composition and arrangement of subtypes I-C and II-A were found to be more stable in *B. pseudocatenulatum*. Subtype I-U in this species showed *csb1* and *csb2* genes adjacent to each other, whereas that in *B. longum* subsp. *longum* 17-1B has been shown to possess *csb2* and *csb3* genes separated from each other (Hidalgo-Cantabrana et al., 2017). In contrast to subtype I-E in *B. longum*, that in *B. pseudocatenulatum* in our study showed an additional element, i.e., the *cse1* gene.

Notably, some incomplete CRISPR-Cas systems have been found. They contained the subtype-specific signature Cas proteins but lacked the Cas proteins essential for immune function (Figure 3B). Strains with incomplete CRISPR-Cas systems of subtype I-C and I-U generally lack the effector complex essential for crRNA maturation (Makarova et al., 2013; Hille et al., 2018). *B. pseudocatenulatum* FGSYC91M2 strain is in lack of Cas2 protein which cooperate with Cas1 protein to capture foreign genetic material, leading to its disability to update enemy blacklist (Wang et al., 2015). *B. pseudocatenulatum* FXJKS15M4 strain possessed neither Cas2 protein nor Cas3 protein, indicating that it cannot recognize infective virus and protect itself. Although these CRISPR-Cas systems were defective, they could be perfectly classified in the phylogenetic trees based on Cas1 and Cas3 amino acid sequences.

The size of CRISPR-Cas systems of each subtype is related to its *cas* gene composition and the number of repeats. Type I systems are generally larger than type II systems because type I systems use multiple Cas protein complexes for interference, whereas type II systems use only Cas9 protein. The sequence length of type I systems, including subtypes I-C, I-U, and I-E, ranges from 9 to 20 kb. Type II-A systems contain fewer *cas* genes and repeats than type I systems, so their sequence length is relatively short (6–9 kb). The location and size of each CRISPR-Cas system are provided in Supplementary Table S2.

The number of repeats in each subtype was found to be different between *B. pseudocatenulatum* strains (Supplementary Figure S1). Subtype I-C presented high variability in the number of repeats, from 2 repeats in the FGSYC76M7 strain to 116 repeats in the FQHXN83M4 strain. The distribution of repeats in subtype I-U was relatively stable at approximately 40 repeats across all strains. Subtype II-A contained the lowest number of repeats, ranging from 13 to 47. The unique I-E subtype, which was present in only the FZJHZ1M1 strain, showed the highest number of repeats at 168.

Pervasive spacer deletion coupled with spacer acquisition have been observed in natural as well as laboratorial conditions (Deveau et al., 2008; Lopez-Sanchez et al., 2012). Such changes in CRIPSR arrays may be explained by environmental selection pressure that drives bacteria to delete the less valuable spacers whilst acquire the more valuable spacers (Horvath et al., 2008). A previous study of *B. longum* found that strains from infant feces possessed lower number of spacers whilst strains from adult feces contained a high number of spacers (Hidalgo-Cantabrana et al., 2017). From this perspective, we speculated that the number of spacers in complete CRISPR-Cas systems (equivalent to the number of repeats) is related to the duration of existence of the strain in the human intestine, i.e., the longer a strain persisted in the human intestine, the more repeats its CRISPR loci contained because of more saved foreign gene fragments. Therefore, we performed a scatter plot to explore the possible correlation between the number of repeats in each *B. pseudocatenulatum* strain and its host’s age. Unexpectedly, we found no correlation between the two variables (Data not shown).

The repeat sequences within each CRISPR-Cas subtype in *B. pseudocatenulatum* were found to be conserved. The length of the repeat sequence was 33 nucleotides in subtype I-C, 29 in subtype I-E and 36 in subtypes I-U and II-A. Notably, the repeat sequences within most subtypes were identical in nucleotide arrangement, except those within subtype I-C, which displayed five nucleotide polymorphisms (Table 1).

### Association Between CRISPR-Cas Systems and Prophages

Spacers in the CRISPR-Cas loci originate from foreign invaders and bacteriophages are the most common threats for bacteria. If a bacterial strain has ever been invaded by a phage, the spacer sequences of the strain may contain a fragment corresponding to the phage genome. Based on this knowledge, we attempted to identify the prophages present in *B. pseudocatenulatum* strains to determine the interaction between this *Bifidobacterium* species and its prophages. In total, 3652 spacer sequences were extracted from the genomes of 41 strains with complete CRISPR-Cas systems. The sequence length ranged from 29 to 42 bp, with 2383 unique base sequences. To further investigate the origins of these foreign DNA fragments, we performed a BLAST search of the extracted spacer sequences against the NCBI virus Refseq database (updated on 2020.3.3). Only *Bifidobacterium* phage PMBT6 was targeted by *B. pseudocatenulatum* spacers, with a total of 10 matches from 5 unique spacers, belonging to 10 different strains, indicating the lack of studies on bifidophages.

**FIGURE 4:**
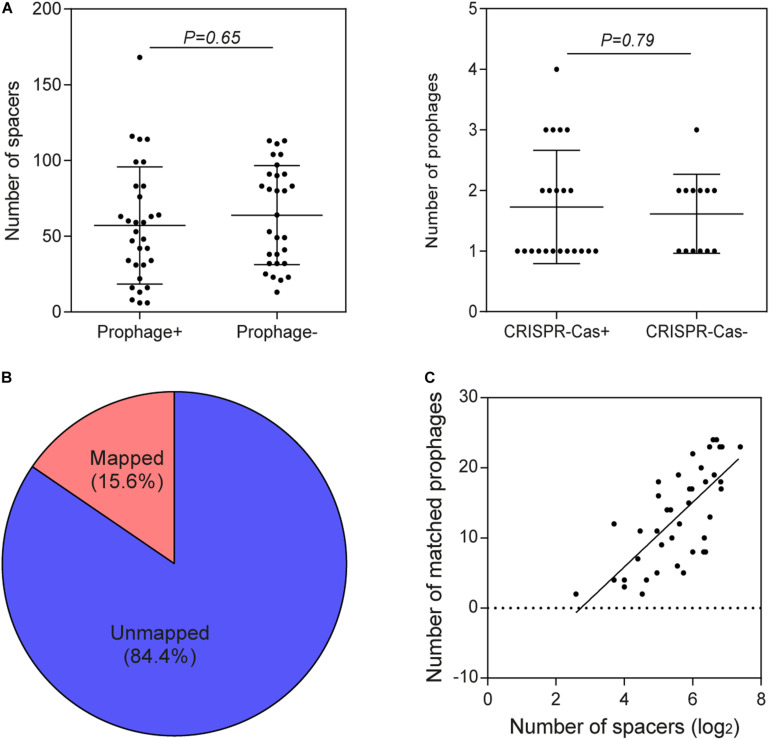
Association between CRISPR-Cas systems and prophages. **(A)** Comparison of the number of spacers in *B. pseudocatenulatum* strains with and without prophages and comparison of the number of prophages in the presence and absence of CRISPR-Cas systems using two-tailed Student’s *t*-test. **(B)** The origin of 15.6% of the spacer sequences was mapped to selected prophages, and that of the remaining spacers could not be matched to any of the integrated prophages or against the RefSeq_viral database. **(C)** CRISPR spacers targeting prophages in *B. pseudocatenulatum* strains. The heat-map represents spacers that matched the prophages in different *B. pseudocatenulatum* strains. The vertical axis represents the selected prophages. The horizontal axis represents the strains carrying CRISPR spacers that target prophages. The color scales represent the number of targeting events, with blue squares representing the absence of matches and red squares representing the highest number of targeting. **(C)** Correlation between the number of spacers in CRISPR arrays and the number of matched prophages (*n* = 41, Spearman’s rank correlation coefficient *r* = 0.7156, *P* < 0.001).

Through a series of screening methods, we investigated 59 prophages in 35 *B. pseudocatenulatum* strains (Table 2). To analyze the association between CRISPR-Cas systems and lysogeny, the strains containing either prophages or CRISPR-Cas systems were evaluated. The results showed that the presence of CRISPR arrays or prophages was not associated with the number of prophage fragments or CRISPR spacers, respectively (Figure 4A). Approximately 15.6% of the bacterial spacer sequences (371/2383, Figure 4B) were completely mapped to the prophages present in previously reported bifidobacterial strains (Ventura et al., 2010; Hidalgo-Cantabrana et al., 2017) or identified in this study. The more spacers a strain containing a CRISPR-Cas system harbored, the greater the number of prophages it would match (Figure 4C), suggesting that such strains possessed a strong immunity. Importantly, in the strains containing both CRISPR-Cas systems and prophages, the number of spacers was found to be irrelevant to whether there was a spacer targeting its own prophage.

**TABLE 2:** List of prophages found in *B. pseudocatenulatum* strains.

Strains	Name	Location	Start	End	Size	ORF	GC content
A13	Bpseuc_1	Scaffold2	227637	244310	16674	27	61.69%
A13	Bpseuc_2	Scaffold8	175	36952	36778	40	59.27%
A13	Bpseuc_3	Scaffold9	1142	36643	35502	53	58.21%
A14	Bpseuc_4	Scaffold4	69117	85946	16830	25	61.59%
FAHBZ9L5	Bpseuc_5	Scaffold13	19	29389	29371	28	59.60%
FAHBZ9L5	Bpseuc_6	Scaffold18	2260	39492	37233	64	58.86%
FAHWH24M2	Bpseuc_7	Scaffold3	77613	99772	22160	29	61.63%
FAHWH24M2	Bpseuc_8	Scaffold3	99844	126153	26310	36	59.94%
FFJND17M1	Bpseuc_9	Scaffold15	19662	44844	25183	23	59.18%
FFJND17M1	Bpseuc_10	Scaffold8	8	37391	37384	58	63.57%
FGSYC11M1	Bpseuc_11	Scaffold11	68	39551	39484	59	58.16%
FGSYC11M1	Bpseuc_12	Scaffold8	6393	56749	50357	50	63.98%
FGSYC13M1	Bpseuc_13	Scaffold11	1110	17254	16145	26	54.89%
FGSYC13M1	Bpseuc_14	Scaffold15	1356	38694	37339	53	55.82%
FGSYC39M1	Bpseuc_15	Scaffold5	2440	24532	22093	40	58.63%
FGSYC39M1	Bpseuc_16	Scaffold5	36799	64284	27486	23	57.52%
FGSYC3M2	Bpseuc_17	Scaffold1	644814	659818	15005	22	58.58%
FGSYC43M1	Bpseuc_18	Scaffold17	610	18948	18339	22	55.45%
FGSYC43M1	Bpseuc_19	Scaffold18	258	19089	18832	34	59.56%
FGSYC43M1	Bpseuc_20	Scaffold6	98257	141985	43729	59	59.57%
FGSYC6M1	Bpseuc_21	Scaffold13	734	20588	19855	31	59.38%
FGSYC6M1	Bpseuc_22	Scaffold8	43823	69065	25243	22	59.16%
FGSYC76M7	Bpseuc_23	Scaffold11	34493	77744	43252	57	59.03%
FGSYC91M2	Bpseuc_24	Scaffold4	41764	59356	17593	27	61.75%
FGSZY20M1	Bpseuc_25	Scaffold15	812	14292	13481	25	58.67%
FJLHD2M3	Bpseuc_26	Scaffold3	19631	45491	25861	21	65.53%
FJLHD33M2	Bpseuc_27	Scaffold7	3832	39993	36162	51	59.68%
FJSNT37M5	Bpseuc_28	Scaffold12	11435	33102	21668	31	61.15%
FNXHL5M2	Bpseuc_29	Scaffold17	1037	36153	35117	56	58.43%
FNXYCHL12M2	Bpseuc_30	Scaffold15	66	19263	19198	35	57.29%
FQHXN112M3	Bpseuc_31	Scaffold11	10551	33925	23375	36	55.32%
FQHXN112M3	Bpseuc_32	Scaffold3	39	30984	30946	52	54.94%
FQHXN5M4	Bpseuc_33	Scaffold11	3043	35653	32611	51	58.72%
FQHXN5M4	Bpseuc_34	Scaffold6	97354	125807	28454	45	59.57%
FQHXN6M4	Bpseuc_35	Scaffold7	20186	45415	25230	22	59.14%
FQHXN72M4	Bpseuc_36	Scaffold6	41789	59417	17629	27	61.77%
FQHXN83M4	Bpseuc_37	Scaffold4	15022	55324	40303	42	56.86%
FQHXN83M4	Bpseuc_38	Scaffold4	55295	72648	17354	22	55.25%
FQHXN83M4	Bpseuc_39	Scaffold4	118715	143733	25019	22	55.95%
FQHXN8M3	Bpseuc_40	Scaffold2	106129	150800	44672	59	58.95%
FQHXN8M3	Bpseuc_41	Scaffold2	145017	162282	17266	25	57.72%
FQHXN8M3	Bpseuc_42	Scaffold9	8469	54895	46427	49	59.53%
FSHXXA2M9	Bpseuc_43	Scaffold9	1804	24122	22319	39	58.53%
FXJWS24M3	Bpseuc_44	Scaffold12	4191	26020	21830	29	55.45%
FXJWS24M3	Bpseuc_45	Scaffold4	61672	119564	57893	82	54.42%
FXJWS24M3	Bpseuc_46	Scaffold4	129990	175070	45081	47	56.27%
FYNDL22M6	Bpseuc_47	Scaffold3	265958	294283	28326	34	63.00%
FYNLJ23M6	Bpseuc_48	Scaffold9	63575	86482	22908	28	56.73%
FZJHZ1M1	Bpseuc_49	Scaffold9	1998	22072	20075	36	58.27%
HuNa38	Bpseuc_50	Scaffold11	23457	56669	33213	51	60.78%
HuNan_2016	Bpseuc_51	Scaffold17	1	26720	26720	47	63.22%
HuNan_2016	Bpseuc_52	Scaffold5	117353	154565	37213	52	60.48%
U2	Bpseuc_53	Scaffold12	4175	29437	25263	35	59.17%
U2	Bpseuc_54	Scaffold16	1708	20682	18975	34	59.60%
V6	Bpseuc_55	Scaffold6	85538	115164	29627	37	58.95%
XZ28R1	Bpseuc_56	Scaffold10	1210	39052	37843	70	54.92%
XZ28R1	Bpseuc_57	Scaffold10	21621	44575	22955	31	54.92%
XZ28R1	Bpseuc_58	Scaffold4	109934	127530	17597	26	61.73%
XZ28R1	Bpseuc_59	Scaffold5	3834	41268	37435	54	59.66%

**FIGURE 5:**
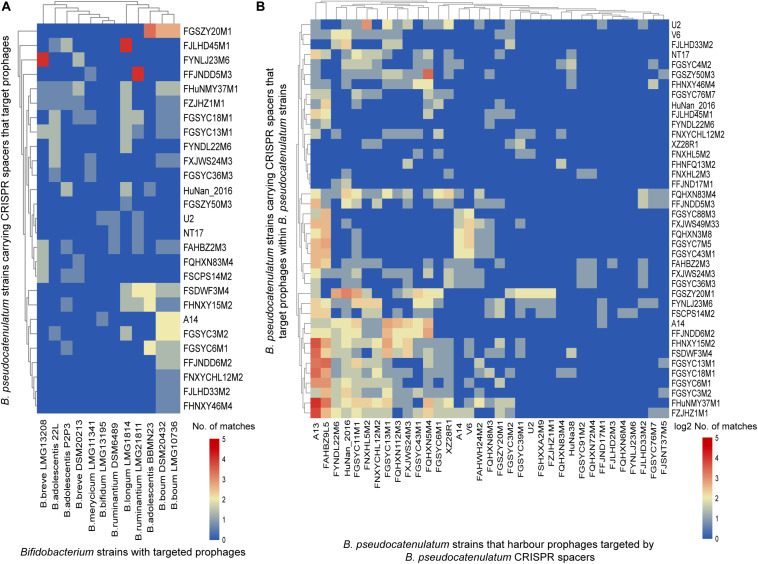
CRISPR spacers targeting prophages in bifidobacterial genomes. **(A)** *B. pseudocatenulatum* CRISPR spacers targeting prophages in other Bifidobacterium strains. The heat-map represents spacers that matched the prophages in different Bifidobacterium strains. The horizontal axis represents other Bifidobacterium strains with targeted prophages. The vertical axis represents the *B. pseudocatenulatum* strains carrying CRISPR spacers that target prophages. The color scales represent the number of targeting events, with blue squares representing the absence of matches and red squares representing the highest number of targeting. **(B)** *B. pseudocatenulatum* CRISPR spacers targeting prophages in strains belonging to its species. The horizontal axis represents *B. pseudocatenulatum* strains that harbor prophages targeted by *B. pseudocatenulatum* CRISPR spacers. The vertical axis represents the *B. pseudocatenulatum* strains carrying CRISPR spacers that target prophages within *B. pseudocatenulatum* strains.

In order to explore the homology between spacers in *B. pseudocatenulatum* and prophages identified in other *Bifidobacterium* strains, a BLAST search of spacers against the prophages in 76 genomes reported before were performed (Lugli et al., 2016; Hidalgo-Cantabrana et al., 2017). Spacers presenting in the 41 complete CRISPR-Cas systems within *B. pseudocatenulatum* showed homology to prophages in other 12 bifidobacterial strains (Figure 5A), indicating *B. pseudocatenulatum* strains acquired immunity against temperate phages which could infect other *Bifidobacterium* species, including *Bifidobacterium boum*, *Bifidobacterium adolescentis*, *Bifidobacterium ruminantium*, *Bifidobacterium breve*, *Bifidobacterium bifidum*, *Bifidobacterium merycicum*, and *B. longum*.

Among the 41 *B. pseudocatenulatum* strains harboring complete CRISPR-Cas systems, 27 strains presented spacers targeting prophages in other *Bifidobacterium* spp. genomes (Figure 5A). The *B. pseudocatenulatum* strains FGSZY20M1 and FSDWF3M4 contained a higher number of spacers matching the prophages in the genomes of other *Bifidobacterium* spp., such as *B. boum*, *B. adolescentis*, which were most frequently targeted by CRISPR spacers of *B. pseudocatenulatum* (Figure 5A). Besides, *B. pseudocatenulatum* strains FHuNMY37M1, FZJHZ1M1, and FGSYC18M1 presented the highest number of spacers targeting prophages detected in this study while FFJND17M1 presented the least (Figure 5B).

Among the prophages detected in *B. pseudocatenulatum* species, Bpseuc_3 and Bpseuc_6 prophages were detected in the genomes of A13 and FAHBZ9L5, corresponding to spacer sequences in 38 and 34 strains, respectively (Figure 5B). In addition, prophages of Bpseuc_26, Bpseuc_35 and Bpseuc_48 within FJLHD2M3, FQHXN6M4, and FYNLJ23M6, presented the most uncommon prophage within *B. pseudocatunulatum* because the spacers matching these prophages can only be found in few strains (Figure 5B). Notably, only *B. pseudocatenulatum* FXJWS24M3 strain displayed a spacer sequence targeting its own prophage Bpseuc_44 (Figure 5B), indicating a potential to prevent prophage induction and lysis as mentioned in previous studies (Edgar and Qimron, 2010; Goldberg et al., 2014).

### Prophages Within *B. pseudocatenulatum* Generally Lack the Genes Essential for the Phage Life Cycle

**FIGURE 6:**
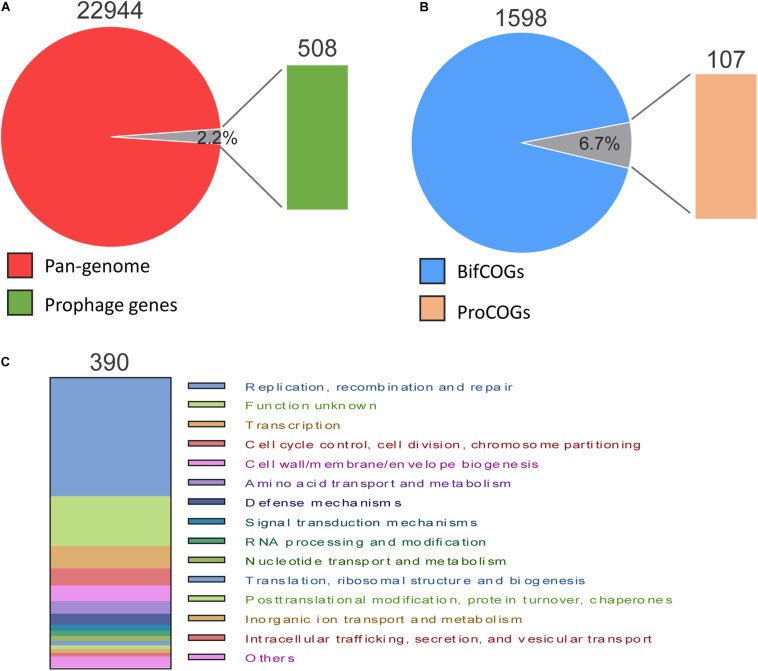
Pan-genome and COG comparison between prophages and *B. pseudocatenulatum* genome. **(A)** Relative proportion of the number of genes in *B. pseudocatenulatum* and prophages. **(B)** Relative proportion of the number of bacterial COGs (BifCOGs) and prophage COGs (ProCOGs). **(C)** Abundance of the ProCOGs with identical predicted functions.

To determine the contribution of prophages to the *B. pseudocatenulatum* genomes, we annotated all genes of both prophages and the bacterial hosts’ genomes using NR and COG databases. In total, 22,944 genes were detected in 35 *B. pseudocatenulatum* strains, 508 of which were derived from prophages, accounting for 2.2% of the total bacterial genes (Figure 6A). The proteins that constitute each COG are assumed to be derived from an ancestral protein with similar or identical functions. The COG database^2^ is a popular tool for functional annotation because of its reliable assignment of orthologs and paralogs and careful manual curation (Tatusov et al., 1997; Galperin et al., 2015). We analyzed the gene clusters between *B. pseudocatenulatum* strain genomes and prophages to clarify the effects of prophages on the gene compositions of those strains. In total, 1598 gene clusters were found in 35 strains (BifCOGs), 107 of which originated from prophages (ProGOGs), thus accounting for 6.7% of the total gene clusters across the studied *B. pseudocatenulatum* strains (Figure 6B).

In total, 390 genes were annotated with clear COG classification in the 59 prophages (Figure 6C), 40.8% (159) of which found to be involved in DNA replication, recombination and repair and 7.7% (30 genes) in cell cycle control, cell division and chromosome segmentation, making the third largest COG. However, the functions of a large number of genes contributed by the prophages could not be identified, indicating a vast scope for further investigation.

**FIGURE 7:**
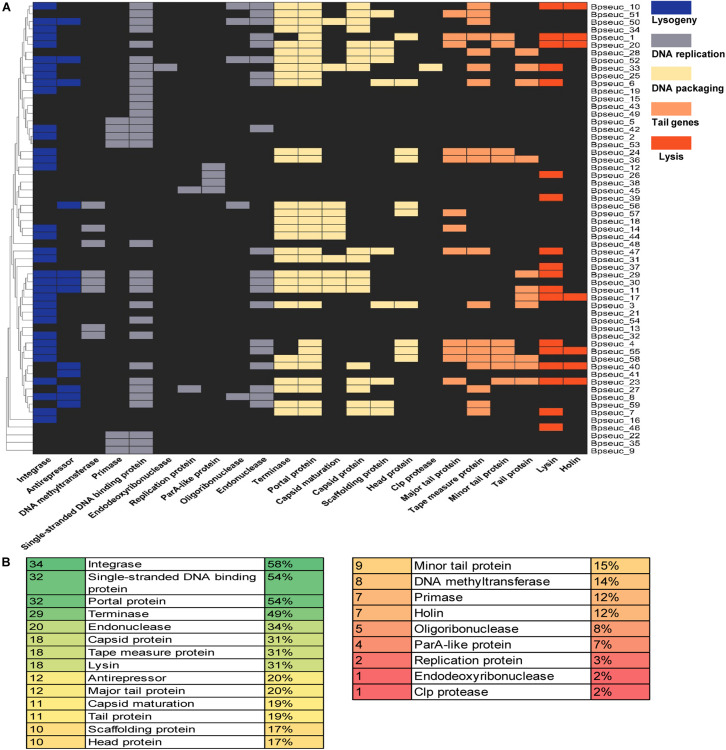
Preservation of genes within the prophages identified based on the genomic functional modules. **(A)** Prophage genes were subdivided in five functional modules supported by a heatmap of the identified genes for each prophage. The prophage names are indicated on the right-hand margin of the heatmap, and the gene names are displayed at the bottom. **(B)** Abundance of individual functions identified within the prophages. The first column shows the number of prophages that encode a particular function listed in the second column, whereas the third column shows the relative percentages.

Database matches allowed a tentative subdivision of the 59 prophages in *B. pseudocatenulatum* genomes into functional modules for a better understanding of their dynamics within their hosts. Based on a previous study (Botstein, 1980), we divided their function into five modules, namely lysogeny, DNA replication, DNA packaging, head and tail morphogenesis, and host lysis (Figure 7A).

Among the identified functional modules, DNA packaging module was well-preserved, with the occurrence of terminase, portal protein and capsid protein genes on 49, 54, and 31% of the prophages, respectively. In contrast, DNA replication was the most variable module, containing fewer preserved genes between the analyzed prophages, most of which lack the protein-encoding genes belong to this module except for the gene encoding the single-stranded DNA-binding protein. Further analysis of individual genes revealed that the most preserved gene was the integrase-encoding gene belonging to the lysogeny module, which was present on 58% of the prophages. The next most preserved genes were the single-stranded DNA-binding protein-encoding gene (54%) belonging to the DNA replication module and the portal protein-encoding gene (54%) belonging to the DNA assembly module (Figure 7B).

### Phylogenetic Analysis of Prophages Within *B. pseudocatenulatum*

**FIGURE 8:**
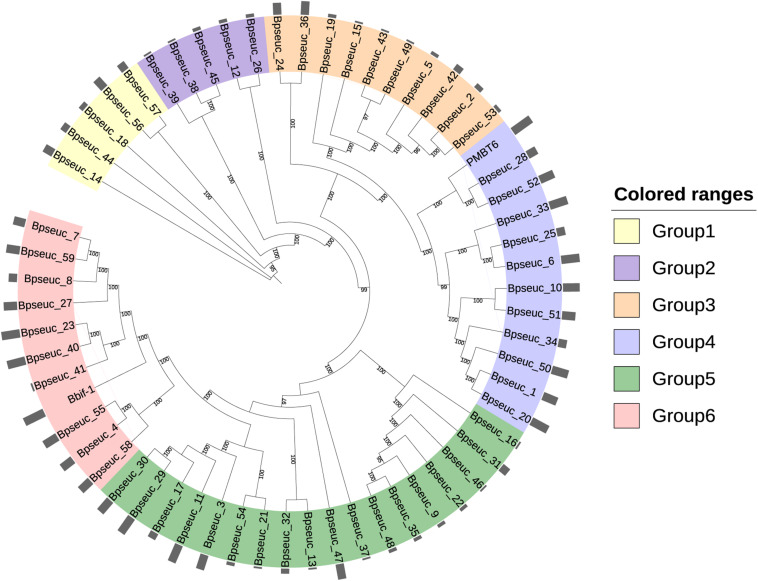
Phylogenetic tree based on the whole genome of prophages. MAFFT was used to perform multiple sequence alignment. The maximum likelihood method was used to construct the phylogenetic tree with 1000 bootstrap replicates. The outermost circle bar represents the number of prophages containing important types of viral functional proteins.

To understand whether the identified prophages were derived from the same origin and homology, we constructed a phylogenetic tree based on its predicted whole genome sequences (Figure 8). In addition to the 59 identified prophages, two bifidophages obtained from the NCBI database, namely Bbif-1 (GCA_002633625.1) and PMBT6 (GCA_006529735.1), were subject to this evolutionary analysis. The homology analysis of these prophages divided them into six groups. The average guanine and cytosine (GC) content of Group1 was 55.3%, of Group2 and Group3 was approximately 59%, and of Group4 and Group6 was variable at around 60%. Group 5 was the most stable group with an average GC content of 57.9%. Comparison of the GC content of the prophages between the groups revealed a significant difference with varying degrees between Group1 and Group3, Group4 and Group6, indicating that the prophages in Group1 were distinctly different from those in the other groups. The average GC content was also significantly different between Group4 and Group5.

A systematic dot plot analysis (Supplementary Figure S2) of the sequences of these 61 *Bifidobacterium* prophages was performed to verify the accuracy of the prophage grouping and highlighted the possible collinearity between these groups. Group1 prophages were the most conserved and shared few homologous genes with those in other groups. Group2 prophages represented an independent prophage population in *B. pseudocatenulatum* as it showed no gene homology within or between the groups. Group3 prophages were relatively conserved and showed partial genome matches with Group5 prophages. Many similar genes were identified between Group4 as well as Group6 prophages. Notably, Group5 represented the most complex group of prophages within *B. pseudocatenulatum* as they showed gene fragment similarities with prophages in each group but the degree of similarity within Group5 was not high. This situation can be explained by pervasive genetic degradation of prophages, characterized by orthologous gene loss after the temperate phage integration into bacterial chromosome (Bobay et al., 2014).

Further, among the genes most shared between these prophages, single-stranded DNA-binding protein encoding genes showed homology between most of the prophage sequences. A homologous gene encoding an unknown protein was also widely distributed in these prophages, suggesting that this protein plays an important function for the prophages. In addition, the genes encoding tape measure protein and integrase were also found to contribute significantly to the complex collinearity between the prophage sequences in *B. pseudocatenulatum*.

## Discussion

*B. pseudocatenulatum* is ubiquitous in the human gut across all ages (Turroni et al., 2012) and has been proven to possess multiple probiotic properties (Yang et al., 2017; Agusti et al., 2018; Peiroten et al., 2019). In this study, we analyzed the CRISPR-Cas systems and prophages within 66 *B. pseudocatenulatum* strains isolated from human and animal feces to clarify the defense and counter-defense struggle between *B. pseudocatenulatum* and its temperate phages.

The genomes of *B. pseudocatenulatum* strains showed broad diversity in their CRISPR-Cas systems (Table 1, 62%), which is higher than that in *B. longum* (38%) (Hidalgo-Cantabrana et al., 2017) and the estimated occurrence of 46% within bacteria in general (Grissa et al., 2007), but slightly lower than that in most other *Bifidobacteria* species (77%) (Briner et al., 2015). Most strains harbored *cas1* genes displayed complete CRISPR-Cas systems that can recognize exogenous DNA fragments and exert immunological effects. Four subtypes, namely I-C, I-U, I-E, and II-A, were detected in our *B. pseudocatenulatum* strains. Subtype I-E, which has been detected as the most prevalent subtype in the previous CRISPR-Cas study on bifidobacteria (Briner et al., 2015), was only found in *B. pseudocatenulatum* FZJHZ1M1 strain in this study (Table 1), suggesting that this subtype is rare in *B. pseudocatenulatum* species. Type II systems are the least common systems in nature (Makarova et al., 2015) and so are in *Bifidobacterium* species (Briner et al., 2015). Moreover, in a previous study on *Bifidobacterium* CRISPR-Cas systems, subtype II-A was found only in *B. bifidum* and *B. merycicum*. Our study showed that subtype II-A is present in *B. pseudocatenulatum*, indicating that this species has a potential for genome editing. Meanwhile, subtype II-C is likely to be absent in this *Bifidobacterium* species (Figure 2). The difference between II-A and II-C systems is that II-A contains an additional Csn2 protein that interacts with other Cas proteins during the integration of spacer sequences (Garneau et al., 2010); the alternative of the Csn2 protein present in II-C is still unknown. Evolutionary research of type II CRISPR-Cas systems suggested that subtype II-A has evolved from subtype II-C, indicating that the *csn2* gene was possibly acquired by the II-A ancestor during evolution (Chylinski et al., 2014). However, the phylogenetic tree based on the core genes of *Bifidobacterium* in a previous study suggested that *B. pseudocatenulatum* appeared earlier than *B. longum* (Sun et al., 2015). Therefore, it is very likely that *B. pseudocatenulatum* obtained the *csn2* gene through horizontal gene transfer.

Some incomplete type I systems, mostly I-E systems (Figure 3B), were found during the detection of CRISPR-Cas subtypes in *B. pseudocatenulatum*. Most CRISPR-Cas systems with the signature *cas* genes of subtype I-E were incomplete. It is reported that the presence of the anti-CRISPR (Acr) protein and Acr-associated protein (Aca) encoded by the prophages could inhibit the normal function of CRISPR-Cas systems (Marino et al., 2018). Notably, possible Acr protein has been found by mapping protein-coding sequences in prophages against the latest Acr protein database (Marino et al., 2018; Yin et al., 2019), whilst one Acr protein and one Aca protein capable of causing an incomplete CRISPR-Cas system were confirmed in the prophage within A13 whose CRISPR-Cas system was defective (Supplementary Table S3). FQHXN112M3 and FZJHZD11M4 strains possessing incomplete CRISPR-Cas systems showed the self-targeting phenomenon, i.e., the presence of other DNA fragments identical to the spacer sequences in the same bacterial genome. Thus, self-targeting phenomenon could also be a reason for the presence of incomplete CRISPR-Cas systems in *B. pseudocatenulatum* strains.

Spacers in CRISPR loci preserve the immunity record of invasive genomic fragments. In this study, *B. pseudocatenulatum* displayed CRISPR spacers targeting prophages not only within its own species but also in other *Bifidobacterium* species (Figure 5), which is in accordance with the results of previous report on *B. longum* (Hidalgo-Cantabrana et al., 2017). The spacers in *B. pseudocatenulatum* matching prophages in other bifidobacterial strains may suggest that those species share the same ecological niche in the human gut. However, the presence of diverse spacers in *B. pseudocatenulatum* supports the prevalence of phages in human gut (Shkoporov and Hill, 2019), especially for the temperate phages (Kim and Bae, 2018). In this respect, CRISPR-Cas systems provide this species with an evolutionary advantage, acting as a strong defense mechanism to avoid prophage predation or other foreign DNA fragments invasion.

Over 50% *B. pseudocatenulatum* strains (Table 2) have prophages. The prevalence of lysogeny is in accordance with that in human gut microbiota (Kim and Bae, 2018) as well as that in aquatic bacteria (Castillo et al., 2018). The contribution of prophages to the bacterial genomes identified in our study was slightly lower than that in a previous bifidophage study (Lugli et al., 2016), probably because the previous study evaluated incomplete prophage fragments, whereas our study focused on complete prophages.

Prophages within *B. pseudocatenulatum* are defective to a large extent. The well preserved integrase-encoding gene was found on only 58% of all *B. pseudocatenulatum* prophages, whereas it has been reported to be present in up to 90% of *Bifidobacterium* prophages (Lugli et al., 2016). The expression of the genes in the host lysis module is essential for the entry of the prophage into the lytic cycle (Hyman and Abedon, 2010), whereas the prophages in our study generally lacked genes encoding lysis-related proteins, indicating that their lytic cycle is unlikely to be induced. In addition, the retention of other important viral functional genes of prophages in *B. pseudocatenulatum* genomes is not as complete as that in other *Bifidobacterium* species. Notably, in the lysogeny module of 11 prophages, we observed the presence of genes encoding putative toxin-antitoxin family proteins, which may be crucial for the stable retention of the prophages in the host cells (Guo et al., 2014; Yao et al., 2018) and for the protection of the hosts against further phage infection (Samson et al., 2013). However, prophage degeneration is a common phenomenon under purifying selection (Canchaya et al., 2003; Asadulghani et al., 2009; Bobay et al., 2014). A study on *E. coli* and *Salmonella enterica* (Bobay et al., 2014) reported that gut bacteria generally have a domestication effect on prophages, characterized by rapid prophage inactivation followed by much slower degradation.

The prophages found in *B. pseudocatenulatum* showed abundant diversity and were divided into six groups by whole genome alignment, phylogenetic tree construction (Figure 8) and collinearity analysis (Supplementary Figure S2), each group representing the possible phage source for this species. Notably, DNA fragments of bacteriophage PMBT6 isolated from other *Bifidobacterium* species were found to be completely consistent with unique five spacer sequences present in 10 strains in our study, whereas no spacer sequence was perfectly matched to the DNA fragments of phage Bbif-1 isolated from *B. bifidum*. This phenomenon indicates that the same bifidophage may invade several host *Bifidobacterium* species/strains. Although phage selection is generally considered to be narrow for the host, increasing evidence suggests that phages have a broad host range in nature (Dekel-Bird et al., 2015; Kauffman et al., 2018). The bacteria that share the same ecological niche (Bono et al., 2013) or have the same outer membrane phage receptor binding proteins (Takeuchi et al., 2016; Dowah and Clokie, 2018) are likely to have the same phage predator. A recent study revealed the effect of phage receptor expression on bacterial susceptibility to phage infection (Castillo et al., 2019).

As a powerful genome editing tool, CRISPR has been receiving much attention. In addition to genome editing, CRISPR-Cas systems have also been proven useful in probiotic research applications. The conservation of CRISPR spacer sequences has enabled the traceability and evolutionary analysis of probiotics (Barrangou and Dudley, 2016), which has been used in *Lactobacillus buchneri* strains genotyping (Briner and Barrangou, 2014) and new species-level taxa identification (Zhou et al., 2020). Meanwhile, the role of prophage in antibiotic resistance genes (ARGs) transfer has been revealed (Haaber et al., 2016) and prophages carrying ARGs were also found in some *Bifidobacterium* strains (Mancino et al., 2019). This study is the first systemic analysis of CRISPR-Cas systems and prophages in *B. pseudocatenulatum*, which may provide insights for classification of this species as a probiotics.

It was also found that the prophages contributed to the genomic diversity in *B. pseudocatenulatum*, accounting for 2.2% in pan-genome and 6.7% in COG (Figure 6). Besides, primed spacer acquisition (Fineran et al., 2014) was also found within this species with several different spacer sequences in a certain CRISPR locus corresponding to the same temperate phage genome (Figure 5B), providing selective pressure for phage evolution and genomic diversity. Future studies are warranted to better understand the interaction between *B. pseudocatenulatum* prophage and its host by exploring the existence of some genetic elements in prophage driving bacterial evolution, or functional gene clusters helping host adaptation to harsh environment, as shown in the previous study on marine bacteria (Castillo et al., 2018). Besides, the isolation of difficult-to-culture phages from culturable bacteria by prophage induction could be used to improve our understanding of the bacteria–phage network (Mavrich et al., 2018).

However, this study still had some limitations. Protospacer adjacent motif (PAM) is a short, conserved sequence and essential for CRISPR target recognition (Hille et al., 2018). The identification of PAM is dependent on the analysis of protospacers, and the spacers sequence in the targeted DNA together with the upstream (5′-end) and downstream (3′-end) region (Gleditzsch et al., 2019). This study failed to determine PAM sequences of different CRISPR-Cas subtypes within *B. pseudocatenulatum* due to the limited number of sequenced *Bifidobacterum* phages genomes. In addition, the temperate phage integration site was not analyzed owing to the gaps presenting in the draft genomes. The main strength of this study is that the identification criterion for prophages was the presence of a complete match between the spacer sequences in CRISPR loci and the prophage, so the possibility of mismatch was extremely low. Furthermore, prophage grouping was subjected to evolutionary and collinear analyses to ensure high reliability. A careful analysis of prophages will help us select strains for prophage induction in the future study.

## Conclusion

This study highlights the coevolution of *B. pseudocatenulatum* and bacteriophages, providing insights into the interaction between them. In this study, *B. pseudocatenulatum* showed the presence of a wide variety of CRISPR-Cas systems to protect itself against the invasion of foreign DNA fragments. The majority of phage DNA fragments (prophages) already inserted into the bacterial host genome were defective in genes associated with the disruption of host cells, which could be explained by purifying selection of temperate phage after its integration into bacterial chromosome (Bobay et al., 2014). Prophages within *B. pseudocatenulatum* tend to be inactive and unlike to enter lytic cycle spontaneously and release virions thereof. Notably, Acr protein and Aca protein encoding genes were found in the prophage from A13 strain presenting incomplete I-U system, which may represent a counter-defense strategy of temperate *Bifidobacterium* phage against CRISPR-Cas system. To further explore the defense–counter-defense strategy between *B. pseudocatenulatum* and its phages, future studies should perform prophage induction and obtain their genomic data.

## Data Availability Statement

The datasets generated for this study can be found in the BioProject, under accession number PRJNA577207.

## Author Contributions

GW, ZL, and WC conceptualized, reviewed and edited the manuscript. GW and QL contributed to data curation, investigation and writing the original draft. GW, QL, ZP, LW, and PT helped with the formal analysis. ZL and WC were responsible for the funding acquisition and project administration. GW, ZL, and HZ worked on the methodology. ZL, JZ, HZ, and WC contributed to resources. ZP, LW, and PT worked on the software. WC supervised. ZL and JZ validated the study.

## Conflict of Interest

ZL was employed by Bright Dairy & Food Co., Ltd. The remaining authors declare that the research was conducted in the absence of any commercial or financial relationships that could be construed as a potential conflict of interest.

## Footnotes

1 https://crisprcas.i2bc.paris-saclay.fr/

2 http://www.ncbi.nlm.nih.gov/COG/

## Supplementary Material

FIGURE S1 Box and Whisker representation of the number of CRISPR repeats detected in the CRISPR loci of each CRISPR subtype.

FIGURE S2 A collinearity dot plot analysis of the 61 *Bifidobacterium* prophages.

TABLE S1 General genomic features of *B. pseudocatenulatum* strains in this study.

TABLE S2 Summary of the CRISPR-Cas locus within bacterial genomes.

TABLE S3 Sequences of Acr and Aca like proteins in prophage belonging to A13.
