# Supplementary figures and images for: The Diversity of the CRISPR-Cas System and Prophages Present in the Genome Reveals the Co-evolution of Bifidobacterium pseudocatenulatum and Phages
Source: Front Microbiol. 2020 May 26;11:1088. doi: 10.3389/fmicb.2020.01088 (PMC7264901; doi:10.3389/fmicb.2020.01088)

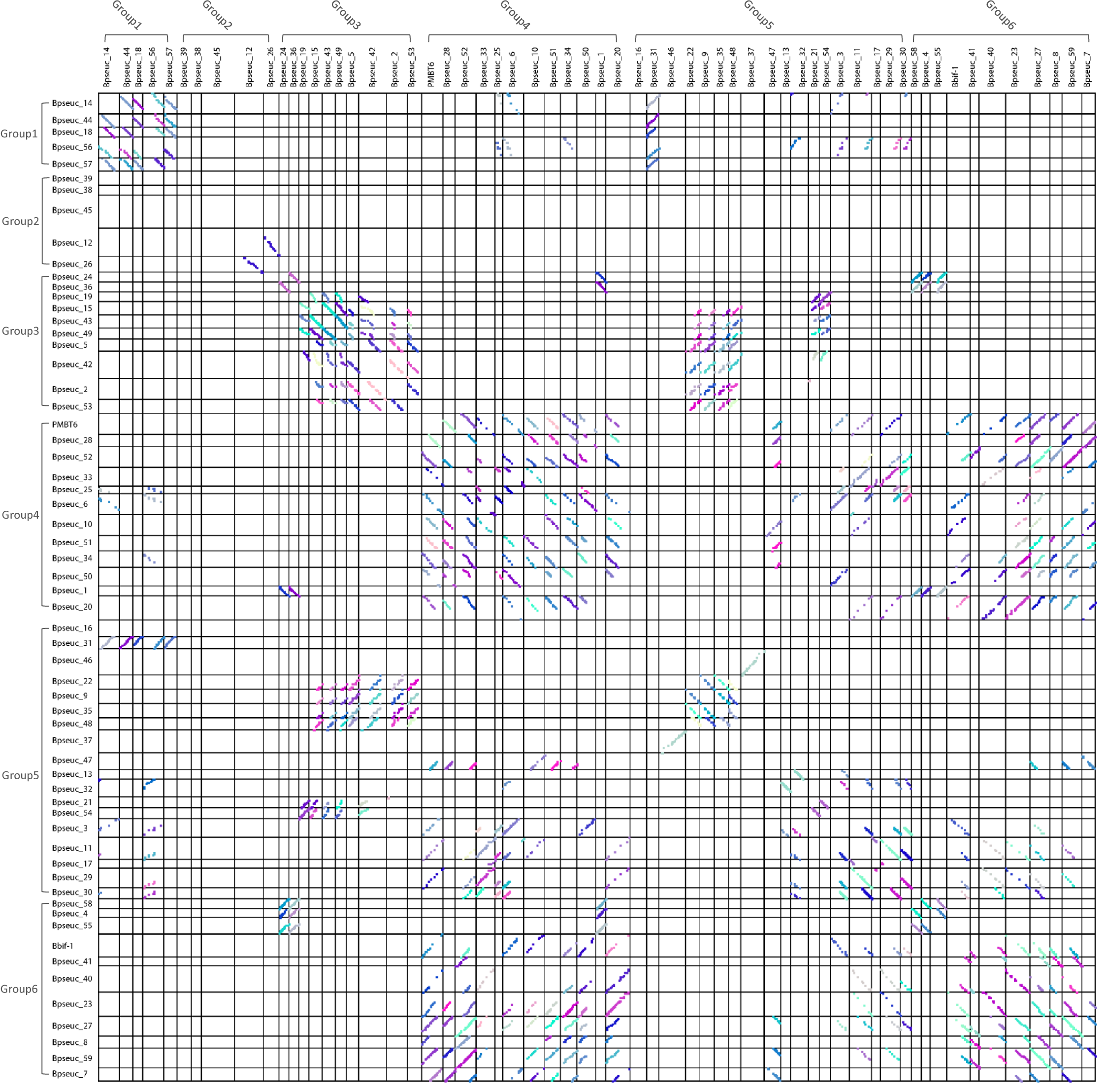

Supplement: FIGURE S2 — A collinearity dot plot analysis of the 61 Bifidobacterium prophages. [file Image_2.TIF]
