# Supplementary material for: The Diversity of the CRISPR-Cas System and Prophages Present in the Genome Reveals the Co-evolution of Bifidobacterium pseudocatenulatum and Phages
Source: Front Microbiol. 2020 May 26;11:1088. doi: 10.3389/fmicb.2020.01088 (PMC7264901; doi:10.3389/fmicb.2020.01088)
Supplement: TABLE S1 — General genomic features of B. pseudocatenulatum strains in this study. [file Data_Sheet_1.docx]

**Table S1.** General genomic features of *B. pseudocatenulatum* strains in this study.

| Strain | Origin | | Age Range | Genome Size(bp) | GC content(%) | No. of ORFs | No. of rRNAs | No. of tRNAs | Accession no. |
| --- | --- | --- | --- | --- | --- | --- | --- | --- | --- |
| V6 | Human | 0-1 | | 2211924 | 56.22 | 2067 | 3 | 56 | SRR10351532 |
| U2 | Human |  |  | 2303685 | 56.62 | 2043 | 3 | 54 | SRR10351533 |
| FZJHZ1M1 | Human |  |  | 2745943 | 55.97 | 2738 | 4 | 60 | SRR10351539 |
| FFJND7M3 | Human |  |  | 2240851 | 56.56 | 2015 | 3 | 55 | SRR10350725 |
| FSDWF3M4 | Human |  |  | 2128625 | 56.38 | 1923 | 3 | 54 | SRR10351546 |
| FFJND17M1 | Human |  |  | 2805417 | 56.87 | 1876 | 2 | 55 | SRR10350726 |
| FSHXXA2M9 | Human |  |  | 2306304 | 56.41 | 2036 | 3 | 53 | SRR10351545 |
| FNXHL2M3 | Human | 1-10 | | 2146412 | 56.57 | 1919 | 2 | 54 | SRR10351549 |
| FHNXY15M2 | Human |  |  | 2215934 | 56.41 | 1964 | 4 | 53 | SRR10350739 |
| FAHBZ9L5 | Human |  |  | 2517672 | 56.51 | 2406 | 3 | 55 | SRR10350738 |
| FGSZY20M1 | Human |  |  | 2164853 | 56.51 | 1942 | 2 | 52 | SRR10350743 |
| HuNa38 | Human |  |  | 2253877 | 56.31 | 1989 | 3 | 56 | SRR10351536 |
| FNMHLBE12M7 | Human |  |  | 2237771 | 56.39 | 1968 | 3 | 55 | SRR10350728 |
| FZJHZD11M4 | Human |  |  | 2371998 | 56.83 | 2105 | 2 | 56 | SRR10351538 |
| FGSZY50M3 | Human | 11-20 | | 2230726 | 56.41 | 1928 | 3 | 55 | SRR10350742 |
| FNXHL5M2 | Human |  |  | 2185745 | 56.31 | 1973 | 3 | 57 | SRR10351548 |
| FHNFQ13M2 | Human |  |  | 2255611 | 56.33 | 2023 | 3 | 55 | SRR10350741 |
| FGSYC76M7 | Human |  |  | 2458900 | 56.22 | 2208 | 3 | 66 | SRR10350748 |
| FGSYC88M3 | Human |  |  | 2173454 | 57.68 | 1819 | 1 | 55 | SRR10350745 |
| FGSYC91M2 | Human |  |  | 2133161 | 57.36 | 1773 | 1 | 54 | SRR10350744 |
| FGSYC87M1 | Human |  |  | 2205047 | 57.55 | 1825 | 3 | 55 | SRR10350746 |
| FHuNMY37M1 | Human | 21-30 | | 2265339 | 56.2 | 1947 | 3 | 53 | SRR10350735 |
| FFJNDD6M2 | Human |  |  | 2232138 | 57.34 | 1867 | 3 | 54 | SRR10350723 |
| FHNFQ3M1 | Human |  |  | 2203012 | 56.52 | 1917 | 3 | 53 | SRR10350740 |
| FQHXN5M4 | Human |  |  | 2237453 | 57.64 | 1931 | 3 | 54 | SRR10351528 |
| FXJWS49M33 | Human | 31-40 | | 2165567 | 57.49 | 1824 | 1 | 56 | SRR10351542 |
| FQHXN3M8 | Human |  |  | 2309103 | 57.47 | 1938 | 1 | 54 | SRR10351529 |
| FQHXN112M3 | Human |  |  | 2327568 | 57.24 | 2022 | 2 | 60 | SRR10351530 |
| FQHXN83M4 | Human |  |  | 2321315 | 57.26 | 1999 | 3 | 63 | SRR10351525 |
| FFJNDD5M3 | Human |  |  | 2238855 | 57.32 | 1817 | 3 | 56 | SRR10350724 |
| FQHXN8M3 | Human |  |  | 2315100 | 57.62 | 1994 | 3 | 58 | SRR10351524 |
| FNXYCHL12M2 | Human | 41-50 | | 2169232 | 56.29 | 1962 | 2 | 54 | SRR10351537 |
| FQHXN72M4 | Human |  |  | 2162129 | 57.49 | 1806 | 3 | 55 | SRR10351526 |
| FXJWS24M3 | Human |  |  | 2462483 | 57.32 | 2201 | 1 | 66 | SRR10351543 |
| FJLHD2M3 | Human |  |  | 2213292 | 57.5 | 1829 | 1 | 55 | SRR10350734 |
| FJLHD45M1 | Human |  |  | 2156228 | 57.15 | 1779 | 3 | 53 | SRR10350732 |
| FGSYC39M1 | Human | 51-60 | | 2240039 | 57.47 | 1884 | 3 | 52 | SRR10350755 |
| FAHWH24M2 | Human |  |  | 2238859 | 56.44 | 2072 | 3 | 52 | SRR10350727 |
| FGSYC43M1 | Human |  |  | 2398878 | 57.59 | 2081 | 2 | 59 | SRR10350753 |
| FGSYC5M4 | Human |  |  | 2153019 | 57.08 | 1778 | 3 | 55 | SRR10350751 |
| FGSYC36M3 | Human |  |  | 2188569 | 57.38 | 1824 | 1 | 56 | SRR10350756 |
| FJLHD4M2 | Human |  |  | 2213520 | 57.5 | 1829 | 3 | 57 | SRR10350731 |
| FGSYC7M5 | Human |  |  | 2211332 | 57.33 | 1853 | 3 | 54 | SRR10350747 |
| FJLHD33M2 | Human |  |  | 2250447 | 57.28 | 1891 | 3 | 48 | SRR10350733 |
| XZ28R1 | Human | 61-70 | | 2331982 | 56.4 | 2121 | 3 | 54 | SRR10351531 |
| FGSYC3M2 | Human |  |  | 2210186 | 57.31 | 1872 | 1 | 54 | SRR10350754 |
| FGSYC6M1 | Human |  |  | 2289408 | 57.71 | 1979 | 5 | 55 | SRR10350750 |
| FGSYC13M1 | Human |  |  | 2247479 | 57.13 | 1950 | 1 | 56 | SRR10350758 |
| FYNLJ23M6 | Human |  |  | 2274284 | 56.58 | 2002 | 3 | 55 | SRR10351540 |
| FGSYC18M1 | Human |  |  | 2225307 | 57.47 | 1898 | 3 | 55 | SRR10350757 |
| FXJKS15M4 | Human |  |  | 2253196 | 56.51 | 2034 | 3 | 53 | SRR10351544 |
| FQHXN6M4 | Human | 71-80 | | 2215259 | 57.38 | 1841 | 1 | 54 | SRR10351527 |
| FGSYC11M1 | Human |  |  | 2283835 | 57.84 | 1962 | 2 | 53 | SRR10350722 |
| FGSYC4M2 | Human |  |  | 2139417 | 57.33 | 1769 | 3 | 55 | SRR10350752 |
| NT17 | Human |  |  | 2224371 | 56.31 | 1989 | 2 | 57 | SRR10351534 |
| FAHBZ2M3 | Human |  |  | 2157897 | 56.22 | 1890 | 3 | 54 | SRR10350749 |
| FHNXY46M4 | Human | 81-90 | | 2156605 | 56.21 | 1897 | 3 | 55 | SRR10350737 |
| FGSYC12M4 | Human |  |  | 2200831 | 57.47 | 1840 | 3 | 56 | SRR10350759 |
| FHuNMY10M3 | Human |  |  | 2231396 | 56.44 | 1970 | 3 | 55 | SRR10350736 |
| FJSNT37M5 | Human |  |  | 2245742 | 57.52 | 1878 | 2 | 54 | SRR10350729 |
| FJSNT36M3 | Human |  |  | 2210383 | 57.27 | 1835 | 2 | 54 | SRR10350730 |
| HuNan_2016 | Human | >90 | | 2223876 | 56.53 | 1980 | 1 | 55 | SRR10351535 |
| A14 | Human |  |  | 2180604 | 56.67 | 1964 | 3 | 54 | SRR10350760 |
| A13 | Human |  |  | 2477035 | 56.82 | 2212 | 2 | 55 | SRR10350761 |
| FSCPS14M2 | Chicken | / | | 2278111 | 56.52 | 2025 | 3 | 53 | SRR10351547 |
| FYNDL22M6 | Cow | / | | 2105392 | 56.64 | 1904 | 3 | 55 | SRR10351541 |

**Table S2.** Summary of the CRISPR-Cas locus within bacterial genomes.

| **Strain** | **CRISPR-Cas subtype** | **Strand** | **Location** | **Start** | **End** | **Length** |
| --- | --- | --- | --- | --- | --- | --- |
|  |  |  |  |  |  |  |
| V6 | I-C | + | whole | 1381900 | 1391920 | 10020 |
| U2 | I-C | - | Scaffold8 | 4664 | 17302 | 12638 |
| FZJHZ1M1 | I-E | + | Scaffold25 | 13024 | 31808 | 18784 |
| FSDWF3M4 | I-C | + | Scaffold3 | 77545 | 94276 | 16731 |
| FFJND17M1 | I-C | - | Scaffold7 | 55 | 9061 | 9006 |
| FNXHL2M3 | I-U | + | Scaffold8 | 65408 | 76591 | 11183 |
| FHNXY15M2 | I-C | - | Scaffold7 | 4640 | 19764 | 15124 |
| FGSZY20M1 | I-C | + | Scaffold3 | 71471 | 93078 | 21607 |
| FGSZY50M3 | I-U | + | whole | 1707991 | 1719343 | 11352 |
| FNXHL5M2 | II-A | + | Scaffold8 | 64810 | 71855 | 7045 |
| FHNFQ13M2 | I-C | - | Scaffold9 | 3608 | 13667 | 10059 |
| FGSYC76M7 | I-C | - | Scaffold12 | 3502 | 14250 | 10748 |
| FGSYC88M3 | I-C | - | Scaffold4 | 3498 | 16613 | 13115 |
| FHuNMY37M1 | I-C | + | Scaffold5 | 77070 | 93454 | 16384 |
| FFJNDD6M2 | I-C | - | Scaffold3 | 151069 | 166317 | 15248 |
| FXJWS49M33 | I-C | + | Scaffold2 | 74648 | 90728 | 16080 |
| FQHXN3M8 | I-C | + | Scaffold3 | 73751 | 87791 | 14040 |
| FQHXN83M4 | I-C | + | whole | 68138 | 84615 | 16477 |
| FFJNDD5M3 | I-C | - | Scaffold3 | 157392 | 172080 | 14688 |
| FQHXN72M4 | I-U | + | Scaffold9 | 76723 | 86511 | 9788 |
| FNXYCHL12M2 | II-A | - | whole | 1790560 | 1800032 | 9472 |
| FXJWS24M3 | I-U | + | whole | 1722070 | 1738113 | 16043 |
| FJLHD45M1 | I-C | + | whole | 67855 | 77899 | 10044 |
| FGSYC43M1 | I-C | + | Scaffold2 | 77866 | 93459 | 15593 |
| FGSYC36M3 | I-U | + | Scaffold 1 | 77436 | 90213 | 12777 |
| FGSYC7M5 | I-C | + | Scaffold1 | 69154 | 84087 | 14933 |
| XZ28R1 | I-C | - | Scaffold8 | 3524 | 13211 | 9687 |
| FJLHD33M2 | I-C | + | Scaffold4 | 76585 | 87549 | 10964 |
| FGSYC6M1 | I-C | - | Scaffold3 | 191716 | 204895 | 13179 |
| FGSYC3M2 | I-C | - | Scaffold1 | 737402 | 754526 | 17124 |
| FYNLJ23M6 | I-C | - | Scaffold5 | 86555 | 100994 | 14439 |
| FGSYC13M1 | I-C | - | Scaffold2 | 159797 | 177596 | 17799 |
| FGSYC18M1 | I-C | - | Scaffold4 | 190799 | 207227 | 16428 |
| FGSYC4M2 | I-U | + | Scaffold2 | 73033 | 86525 | 13492 |
| NT17 | I-U | - | Scaffold1 | 640410 | 652720 | 12310 |
| FAHBZ2M3 | I-C | - | Scaffold5 | 82395 | 98782 | 16387 |
| FHNXY46M4 | II-A | - | whole | 1640946 | 1648057 | 7111 |
| HuNan_2016 | II-A | + | Scaffold3 | 70878 | 80172 | 9294 |
| A14 | I-C | - | Scaffold7 | 4637 | 19064 | 14427 |
| FYNDL22M6 | I-C | + | Scaffold5 | 4531 | 14469 | 9938 |
| FSCPS14M2 | I-U | - | whole | 1089250 | 1101576 | 12326 |

**Table S3.** Sequences of Acr and Aca like proteins in prophage belonging to A13.

| **Description** | **Sequence** |
| --- | --- |
| Bpseuc_2_Acr_like_protein | MTFGSKAAFRAARERCGISQKMLADRFGNAVMTVKRWEKPGEAEPPADVQAWLEHMLSQH VEAVEAALDAVDGIEEVQGNPPDHVDLTYYRSQAHYDEFGRDEAVYSVVNARSREIGAIL QAEGYTVRYVYPEDADTVSTLGGRDVQA |
| Bpseuc_2_Aca_like_protein | MGLRSMREGVGLSQQDLCRAIGSSTVGRVWAWEAWSDSPRPKSARDPHLMGLSTAKVLAD ELGMTLDDFWAGLDG |
